# Bridging the gap: Community insights on effectively sharing research findings with the public

**DOI:** 10.1017/cts.2026.10717

**Published:** 2026-03-09

**Authors:** Jennifer Mary Poger, Jessica Abrams Schrodel, Paula C. Moodie, Candace Bordner, Nehath Sheriff, Jennifer B. McCormick, Jennifer L. Kraschnewski

**Affiliations:** 1 Department of Medicine, Penn State College of Medicine, USA; 2 Department of Public Health Sciences, Penn State College of Medicine, USA; 3 Department of Humanities, Penn State College of Medicine, USA

**Keywords:** Lay dissemination, communicating science, research results, translation, engagement

## Abstract

**Introduction:**

Funders increasingly emphasize the ethical imperative to return research results, yet researchers often lack training and clear strategies for effectively sharing findings with lay audiences. While publishing in academic journals is standard practice for dissemination, little guidance exists on translating findings for communities, particularly in rural areas. This qualitative community-guided pilot project aimed to explore and strengthen strategies for sharing study results in accessible ways.

**Methods:**

The Penn State Clinical and Translational Science Institute conducted six semi-structured focus groups in Fall 2023 with geographically dispersed Pennsylvanians. Focus groups introduced participants with and without prior research experience to evidence-based and novel dissemination methods – such as lay summaries and data walks – to gather feedback on preferences and experiences. Data were coded and analyzed using MAXQDA, achieving strong interrater reliability (kappa > 0.70). Themes were developed inductively.

**Results:**

Focus group participants (*N* = 45) were predominantly women (*N* = 39, mean age = 56); 10% identified as Black/African American. Geographically, 49% were rural, 44% suburban, and 7% urban. Major themes included lack of effective communication in the research process, poor representation, and limited access to results. Most participants had never received study findings. Participants preferred receiving easy-to-understand summaries shared by individuals with established community relationships. They also found data walks, where researchers bring key findings printed on posters to community events, to be especially engaging and valuable.

**Conclusion:**

Community-informed dissemination approaches can increase research transparency, engagement, and results translation in communities, particularly in rural areas where accessibility is limited.

## Introduction

Translating research findings into formats that are accessible and meaningful to lay audiences is an essential but often overlooked step in the research process [1–3]. Participants who contribute their time, data, and lived experiences both desire and deserve to understand the outcomes of the studies they support [4,5]. This promotes transparency and continued community engagement in research [3,6–9]. Additionally, communicating research findings supports clinical and translational science by catalyzing evidence-informed change to practice and policy [10–13].

As part of their mission, Clinical and Translational Science Awards aim to “develop, demonstrate and disseminate scientific and operational innovations that improve the efficiency and effectiveness of clinical translation from identification to first-in-human studies to medical practice implementation to community health dissemination” [14]. Ethical frameworks and funder guidelines have underscored the importance of respecting participants throughout the research process, including returning study results [8]. For example, the World Medical Association’s Declaration of Helsinki emphasizes the ethical obligation to publicly share research results [15,16]. Similarly, the Patient-Centered Outcomes Research Institute (PCORI) emphasizes that returning overall results to participants honors their contributions and fulfills a fundamental ethical responsibility [17,18].

Despite these imperatives, dissemination (the act of identifying target audiences and intentionally communicating study results) remains inconsistently practiced, particularly in formats digestible to non-scientific audiences [19–21]. Most researchers focus on traditional scholarly dissemination (e.g., manuscripts, conferences), which often exclude the participants who made the research possible. Research participants may lack access to or knowledge of academic publications, and even with access, may be limited in understanding the technical content [22]. An extensive cross-sectional survey by Schroter et al. (2019) of over 1800 clinical trial authors found that fewer than half of researchers had shared or planned to share study results with participants, and less than a quarter disseminated lay summaries [23]. Researchers cited multiple barriers to disseminating results to participants, including assumptions about interest or comprehension level of participants, as well as their own lack of training or funding to do so. Of note, a minority of research funders (17%) and ethical review boards (18%) suggested dissemination to participants as a critical step in the research process. Although researchers acknowledge the moral importance of sharing results (e.g., promoting trust, empowering participants, and encouraging future research participation), logistical challenges, lack of early planning, and academic culture remain major documented barriers [23–25].

Challenges in disseminating study results is compounded by participant rurality, underscoring the importance of targeted efforts within rural communities to advance clinical and translational science. Failing to return results can weaken relationships between communities and academia, often resulting in a reduced willingness to participate in future research, particularly among rural communities where research accessibility is already limited [26–30]. In contrast, sharing results fosters stronger community-academic partnerships and empowers individuals to make informed healthcare decisions [31,32]. When community members can apply research results to their own lives, it can positively shift their perceptions of research and increase their interest in future involvement [9,31,33]. Additionally, access to research findings equips communities with evidence-based guidance, helping them sift through competing sources of health information and take into consideration science-backed recommendations [23].

Although benefits of lay dissemination are promising, there is limited research on effective strategies for communicating findings to lay audiences or on which formats are most valued by the community [31,32,34,35]. This qualitative pilot study aims to address the gap between funder expectations and real-world dissemination practices by identifying community-preferred approaches to receiving study results. To better understand effective dissemination strategies, our team conducted focus groups with active research participants and the broader community. Findings can inform scalable models that improve access to research outcomes, particularly in rural communities, making lay dissemination an integral and achievable part of the research lifecycle.

## Methods

The Community Engagement team, part of the Penn State Clinical and Translational Science Institute (CTSI), conducted semi-structured focus groups in the Fall of 2023 among geographically dispersed Pennsylvania communities. Broad community members, stakeholders representing constituents across multiple counties in Pennsylvania, and past/current participants of Penn State research were recruited to engage in one of six focus group sessions.

### Recruitment and outreach

Focus group participants were recruited through existing community-academic partnerships within the CTSI, research participant volunteer repositories, and the Penn State Lion Mobile Clinic, which has extensive reach in rural communities (unpublished data). The study was advertised through email invitations, website postings, flyers, and marketing channels within CTSI and broader Penn State communications to enhance recruitment.

### Human participant protections

This qualitative study received an IRB exemption determination. Interested participants contacted the Community Engagement team and were emailed the informed consent document. Attendance at the one-time focus group was implied consent. The research involved no more than minimal risk to participants, and all qualitative interview responses were optional. All data remained de-identified during transcription, coding, and thematic analysis.

### Focus group methodology

The focus groups aimed to engage community members and stakeholders in real-time dialogue to identify ways to improve current research dissemination practices. A 10-question semi-structured interview guide was developed through previously cited themes around research engagement and results dissemination [2,32]. Focus groups also included presentation of evidence-based and novel approaches to lay results dissemination, including lay briefs, newsletters, and data walks [36], to elucidate experiences with, and preferences for, receiving research study results. These were previously co-developed with community partners on prior funded work. The lay briefs consisted of a concise, two-sided, one-page summary highlighting key study results using engaging tables and graphics to enhance understanding. Newsletters included a more expansive report of study progress and research findings and also offered other patient-centered resources related to the study topic. The data walk methodology was introduced through photographs from an actual pilot event that depicted community partners and researchers interacting and discussing large-format infographic posters.

Focus groups were offered both virtually via Zoom and in-person in accessible, frequented locations within the community to help address transportation challenges common in rural areas. Transcriptions were completed by trained members of the research team. To ensure accuracy and reliability, each transcript underwent a two-step data quality check: initial transcription was followed by independent verification by a second transcriber. Names and contact information of study participants were collected separately in a post-survey form via REDCap, a secure web application for survey data collection, in order to issue $50 ClinCard compensation for focus group participation [37]. All identifying information was collected solely for compensation purposes, and the focus group data remained deidentified.

**Figure 1.**
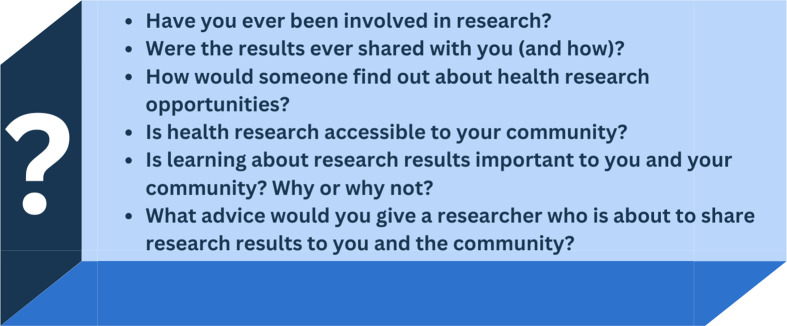
Semi-structured focus group questions.

Focus group facilitators elicited feedback on participant views of research involvement and the return of study findings (Figure 1). Specific conversation starters included views on research, prior experience as a research participant (across the continuum of study type, from qualitative research to drug and device trials), and thoughts on various lay dissemination methods presented (e.g., newsletters, infographics, lay briefs, data walks).

### Theoretical framework

This study was informed by adult experiential learning theory, which emphasizes that adults learn best through a cyclical process of experience, reflection, conceptualization, and experimentation [38]. The design of our dissemination activities and focus group questions were intentionally aligned with this framework. For example, participants were invited to reflect on their past experiences with research (concrete experience and reflective observation), offer interpretations and preferences regarding how results should be shared (abstract conceptualization), and suggest practical ideas for improving dissemination in their communities (active experimentation). By grounding our approach in experiential learning, we aimed to cultivate a space where participants could actively shape guidance for future research dissemination strategies through their lived experience.

### Qualitative analysis

Qualitative analysis was performed by the Qualitative & Mixed Methods Core of Penn State CTSI to extract emerging themes from the transcribed data. Qualitative data were analyzed using MAXQDA software, and kappa coefficient was calculated for interrater reliability (IRR). To ensure consistency and reliability in the coding process, two research team members independently coded the data and achieved strong interrater reliability (kappa > 0.70). Any discrepancies were discussed and resolved through consensus. Codes and themes were developed inductively.

## Results

### Descriptives of sample

Table 1 summarizes demographic characteristics of focus group participants. Among the 46 participants, most were aged 55 years or older (63%), female (87%), and White (86%). Nearly all identified as not Hispanic or Latino (98%). Participants were primarily from rural (48%) and suburban (44%) counties, with few from urban areas (7%). Most held a bachelor’s degree or higher (78%) and reported a broad range of household incomes. Over half (61%) had prior research experience. Demographic patterns were largely consistent across rurality groups, although rural participants tended to be older and exclusively female.

**Table 1.** Demographics of focus group participants

Characteristic	Overall *N* = 46*^1^*	County status		
		Rural *N* = 22 (49%)*^1^*	Suburban *N* = 20 (44%)*^1^*	Urban *N* = 3 (7%)*^1^*
**Age group** (*N* = 46)				
25–34	5 (10.9%)	3 (13.6%)	1 (5.0%)	1 (33.3%)
35–44	7 (15.2%)	2 (9.1%)	5 (25.0%)	0 (0.0%)
45–54	5 (10.9%)	0 (0.0%)	4 (20.0%)	0 (0.0%)
55–64	11 (23.9%)	4 (18.2%)	5 (25.0%)	2 (66.7%)
65 and over	18 (39.1%)	13 (59.1%)	5 (25.0%)	0 (0.0%)
**Race (multiple choice)** (*N* = 44)				
American Indian or Alaskan Native	0 (0.0%)	0 (0.0%)	0 (0.0%)	0 (0.0%)
Asian	2 (4.5%)	0 (0.0%)	1 (5.3%)	1 (33.3%)
Black or African American	4 (9.1%)	1 (4.5%)	3 (15.8%)	0 (0.0%)
Native Hawaiian or other pacific Islander	0 (0.0%)	0 (0.0%)	0 (0.0%)	0 (0.0%)
White	38 (86.4%)	21 (95.5%)	15 (78.9%)	2 (66.7%)
Other	0 (0.0%)	0 (0.0%)	0 (0.0%)	0 (0.0%)
Prefer not to answer	0 (0.0%)	0 (0.0%)	0 (0.0%)	0 (0.0%)
**Ethnicity** (*N* = 43)				
Hispanic or Latino	1 (2.3%)	0 (0.0%)	1 (5.3%)	0 (0.0%)
Not Hispanic or Latino	42 (97.7%)	21 (100.0%)	18 (94.7%)	3 (100.0%)
**Gender Identity** (*N* = 45)				
Female	39 (86.7%)	22 (100.0%)	14 (70.0%)	3 (100.0%)
Male	6 (13.3%)	0 (0.0%)	6 (30.0%)	0 (0.0%)
**Education** (*N* = 45)				
High school graduate	2 (4.4%)	2 (9.1%)	0 (0.0%)	0 (0.0%)
Trade/technical/vocational training	1 (2.2%)	1 (4.5%)	0 (0.0%)	0 (0.0%)
Some college credit with no degree	5 (11.1%)	2 (9.1%)	2 (10.0%)	1 (33.3%)
Associate’s degree	3 (6.7%)	2 (9.1%)	1 (5.0%)	0 (0.0%)
Bachelor’s degree	13 (28.9%)	6 (27.3%)	6 (30.0%)	1 (33.3%)
Master’s degree	13 (28.9%)	6 (27.3%)	7 (35.0%)	0 (0.0%)
Doctorate degree	6 (13.3%)	2 (9.1%)	3 (15.0%)	1 (33.3%)
Medical degree	2 (4.4%)	1 (4.5%)	1 (5.0%)	0 (0.0%)
**Household income** (*N* = 44)				
$0 to $19,999	2 (4.5%)	2 (9.5%)	0 (0.0%)	0 (0.0%)
$20,000–$49,999	9 (20.5%)	6 (28.6%)	2 (10.0%)	1 (33.3%)
$50,000–$89,999	12 (27.3%)	5 (23.8%)	5 (25.0%)	2 (66.7%)
$90,000–$129,999	6 (13.6%)	4 (19.0%)	2 (10.0%)	0 (0.0%)
$130,000–$149,999	2 (4.5%)	0 (0.0%)	2 (10.0%)	0 (0.0%)
$150,000+	9 (20.5%)	2 (9.5%)	7 (35.0%)	0 (0.0%)
Prefer not to answer	4 (9.1%)	2 (9.5%)	2 (10.0%)	0 (0.0%)
**Prior research experience** (*N* = 43)				
Yes	26 (60.5%)	11 (52.4%)	13 (68.4%)	2 (66.7%)
No	17 (39.5%)	10 (47.6%)	6 (31.6%)	1 (33.3%)

1 Values are *n* (%). Percentages are calculated within each rurality category (Rural, Suburban, Urban) based on the number of respondents for each item. Not all participants answered every question.

### Emerging themes

**Figure 2.**
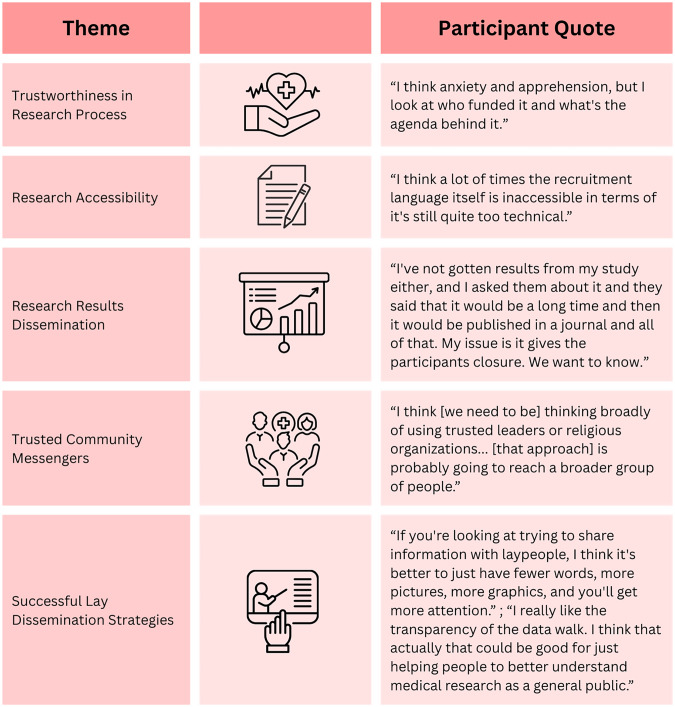
Thematic analysis.

Focus group participants described a range of experiences with research participation primarily in low-risk research studies (focus groups, surveys, behavioral interventions), most of which were positive. Many had participated in studies throughout their lives and cited financial incentives, curiosity, contributing to science, and access to treatment as key motivations. Participants reflected on how research involvement made them feel, often expressing pride and a sense of being *“part of something important”* or contributing to medical breakthroughs. While most reported favorable experiences, they also acknowledged barriers such as time constraints, scheduling conflicts, and travel demands. Recruitment strategies were discussed briefly, whereas barriers to participation and community attitudes toward research were explored in greater depth. Several participants described feelings of exclusion or skepticism toward research within their communities, noting that their involvement in research is often viewed as a low priority by researchers. This was particularly true among the rural community members; *“[A] barrier that I see, the cities get the attention and get the funding and the rural areas do not. So, the constant [perceived] reminder that the rural areas have maybe proportionately less problems, but they still have the same problems.”* Key themes are summarized in Figure 2.

Thematic analyses revealed concerns about the research process, including a perceived lack of transparency and representation, as well as cultural (language; values and belief systems) and geographic barriers that limit both study participation and access to findings. Most participants who had previously engaged in research reported never receiving results from prior studies. As one participant shared, *“Well actually [of] all of the things I participated in, I never received the results. I always wondered, but I never heard anything back. It was like you participate and that’s it. You don’t hear anything else. And it’s kind of frustrating. You want to know…[but] you participate and that’s it.”* Another participant from a rural community had participated in over 7 studies and did not receive results.

Participants also emphasized that traditional research language, especially in recruitment materials, often felt overly technical or inaccessible. *“The research is so hard to digest. You get a white paper that is written at the PhD level and you’re trying to share that with maybe people that are at an eighth or 10th grade reading level and it’s difficult,”* one individual noted, underscoring the importance of plain language for all research communications.

Concerns about research motivations and perceived bias were also raised. One participant reflected, *“I think [it is] anxiety and apprehension, but I look at who funded it and what’s the agenda behind it,”* highlighting a desire for transparency around the purpose and funding of studies. Additionally, general feelings of distrust in research were noted by stakeholder; *“Distrust. Whether it’s with the system or how the research offer came through if it was a spam, but just even if it’s from a physician’s practice, I’ve had a lot of patients tell me, ‘I don’t trust them. I’m not giving them any information.”*

When considering how results should be shared, participants favored materials that are visually engaging, concise, and presented in familiar data visual formats. Infographics and plain-language briefs were preferred over traditional manuscripts. *“I feel like the information, [on the lay brief] is very bright. It’s inviting and then it’s short, it’s sweet. But if they wanted to get more information on anything that’s inside of there, I think a QR code will help*.” Strategies like data walks, where researchers share findings directly within community spaces, were particularly preferred [36]. As one participant noted, *“I think [a] Data Walk is a really good concept for engaging with people and then also showcasing the results to a broader audience.”*

In terms of delivery, participants recommended involving trusted community figures to help share findings more broadly and meaningfully. One suggestion was to engage familiar leaders, such as those of faith-based or local organizations, with one participant explaining, *“Thinking broadly of using trusted leaders or religious organizations… [that approach] is probably going to reach a broader group of people.”*

## Discussion

This qualitative study sought to better understand community preferences for results dissemination, including format, timing, and delivery methods. The ultimate goal was to inform guidance for research teams on how to share findings through community-driven approaches that improve the accessibility and real-world impact of research, uplifting lay dissemination as a central component of translational science.

Unsurprisingly, the majority of participants reported that they had never received results from prior research they were involved in, highlighting a persistent gap in the research-to-community feedback loop. This is especially true in rural communities that already face limited access to research opportunities [26–28]. Focus group results demonstrated a clear desire among participants to receive study results in accessible, easy-to-understand formats. Specifically, lay briefs were preferred over newsletters for their ease of viewing and succinct sharing of information. The in-person design of a data walk held in community spaces appealed to focus group participants to increase translation and engagement. These approaches were seen as more person-centered and relevant than traditional academic formats.

Our findings add to the growing consensus that dissemination should be co-designed with communities and tailored to their specific preferences in order to generate the most impact. Participants in this study revealed themes substantiated by prior work, including the importance of transparency, utilization of plain language, application of cultural relevance, and participant choice in how study results are shared [2,35]. In a 2023 qualitative study of PCORI-funded investigators and patient partners, Sgro and colleagues explored common approaches and barriers to returning aggregate results to study participants [35]. Investigators most often used plain-language summaries (44%), infographics or pictographs (39%), and newsletters (39%) to communicate findings. These formats were typically delivered through email or patient portals, and many were supplemented by videos, community presentations, or mailed materials tailored to participants’ preferences and access needs. Patient partners interviewed emphasized the importance of concise, visually engaging materials and stressed that information should be presented in a way that acknowledges participants’ lived experiences, which resonated in our current findings.

The present study also underscored the value of involving patients and caregivers in designing the materials and selecting dissemination channels, ensuring that results feel relevant and accessible. This includes community members providing feedback on overall content and design, appropriate methods for distribution, and target audience. One effective way to support person-centered dissemination strategies is the utilization of Community Engagement Studios, a one-time, cost-effective model developed through the Clinical and Translational Science Awards (CTSA) program at Vanderbilt University to gather structured feedback from community members [39]. By engaging community partners through these studios, researchers can strengthen study practices and enhance the communication of study findings by incorporating community input into the development, conduct, and dissemination of research. While barriers such as lack of institutional support, delayed IRB approvals, and limited post-study funding remain significant to dissemination goals, planning early and embedding dissemination into study milestones helped researchers overcome these challenges [35]. Requiring lay dissemination plans as part of the initial IRB approval process could facilitate better adherence to federal mandates.

Despite increasing interest in this area, very few academic institutions have formal policies or infrastructure to support lay dissemination [40]. This lack of broader policy support leaves many research teams without the training or resources needed to do this work effectively [24]. However, this also offers an opportunity to the Clinical and Translational Science Award program, given the focus on community engagement and workforce development. Cunningham-Erves et al. (2021) highlights the importance of providing formal training to researchers on how to design and implement dissemination plans. Their evaluation results showed that even brief training interventions can significantly improve researchers’ confidence and intent to engage communities in meaningful ways [31]. More research is needed in this space to establish formal guidance for researcher training and to evaluate its uptake and impact on translational efforts.

Our study contributes to this growing field by providing community-informed methodologies for results dissemination. Focus group members emphasized not only preferred formats but also how and when they wanted to receive information. Themes around consistent communication, ideally through trusted messengers within their own communities, were identified. These insights are reflected in recent literature. For example, McLoughlin and Martinez (2022) argue that dissemination should be seen as an ongoing, two-way exchange, not just a final step, and often research process afterthought [41]. Similarly, projects like QuitSMART Utah and Erie Family Health Centers show how dissemination can be operationalized effectively when it is built into research planning from the start [42,43]. QuitSMART Utah embeds dissemination and implementation strategies into tobacco cessation interventions across diverse community and clinical settings, ensuring that findings are rapidly translated into practice. The Erie Family Health Centers project demonstrates how community health centers can use stakeholder engagement and tailored communication strategies to share research findings with patients and providers in accessible, actionable formats.

Improving how research results are returned back to study participants and the broader public is not only a matter of ethics or transparency but is central to building lasting community partnerships and improving health outcomes. Moving forward, we will work with CTSI-affiliated research teams to bolster education and training around communicating research findings broadly to support the inclusion of community-guided lay dissemination strategies in both ongoing and future projects.

### Limitations

Our research findings reflect the experiences and preferences of participants living in Pennsylvania, many of whom were recruited through existing rural partnerships with community organizations and internal networks. Most participants were White non-Hispanic/Latino, limiting the generalizability of barriers and facilitators to results dissemination across broader community settings. Understanding effective strategies for lay dissemination among underrepresented populations is needed to ensure the broadest reach of research discoveries. Further, participants may also have preferences for additional dissemination formats not presented during the focus groups, such as podcasts, comic strips, or artwork, that could serve as viable tools for communicating research results to lay audiences. Future research exploring the intersection of the arts and health communication in research translation is warranted.

## Conclusion

This study offers valuable insight into how participants engage with research dissemination and highlights practical recommendations directly from community members that can help guide future efforts to improve how research results are shared. Sharing study results in ways that are clear and meaningful to participants and communities helps increase research engagement and improve health knowledge. Developing evidence-based, plain-language dissemination methods can strengthen these efforts and support the growth of informed, research-ready communities. Whether through infographics, presentations, or locally tailored strategies, dissemination is most effective when it is planned with the communities that research is intended to benefit. Dissemination of research findings should be treated as a shared responsibility that helps strengthen trust, deepens engagement, and ensures that research outcomes are returned to the people and communities who make them possible.
